# Dynamic handgrip exercise for the evaluation of mitral valve regurgitation: an echocardiographic study to identify exertion induced severe mitral regurgitation

**DOI:** 10.1007/s10554-020-02063-5

**Published:** 2020-10-16

**Authors:** Mhd Nawar Alachkar, Annemarie Kirschfink, Julian Grebe, Mohammad Almalla, Michael Frick, Andrea Milzi, Wiebke Moersen, Michael Becker, Nikolaus Marx, Ertunc Altiok

**Affiliations:** 1 Department of Cardiology, Angiology and Intensive Care, University Hospital RWTH Aachen, Aachen, Germany; 2 Department of Cardiology, Nephrology and Intensive Care, Rhein-Maas Hospital, Wuerselen, Aachen, Germany

**Keywords:** Handgrip, Mitral valve regurgitation, Echocardiography

## Abstract

Handgrip exercise (HG) has been occasionally used as a stress test in echocardiography. The effect of HG on mitral regurgitation (MR) is not well known. This study aims to evaluate this effect and the possible role of HG in the echocardiographic evaluation of MR. 722 patients with MR were included (18% primary, 82% secondary disease). We calculated effective regurgitant orifice area (EROA) and regurgitant volume (RVOL) at rest and during dynamic HG. Increase in MR was defined as any increase in EROA or RVOL. We analyzed the data to identify possible associations between clinical or echocardiographic parameters and the effect of HG on MR. MR increased during dynamic HG in 390 of 722 patients (54%) (∆EROA = 25%, ∆RVOL = 27%). Increase of regurgitation occurred in 66 of 132 patients with primary MR (50%) and in 324 of 580 patients with secondary MR (55%). This increase was associated with larger baseline EROA and RVOL, but it was independent from other clinical or echocardiographic parameters. In secondary MR, dynamic HG led to a reclassification of regurgitation severity from non-severe at rest to severe MR during HG in 104 of 375 patients (28%). There was a significant association between this upgrade in MR classification and higher New York Heart Association (NYHA) class (OR 1.486, 95%-CI 1.138–1.940, p = 0.004). Dynamic HG exercise increases MR in about half of patients independent of the etiology. Dynamic HG may be used to identify symptomatic patients with non-severe secondary MR at rest but severe MR during exercise.

## Background

Handgrip exercise (HG) is a simple bedside maneuver to increase the intensity of murmur of mitral regurgitation (MR) during auscultation [1]. In addition, HG has been used in echocardiography as a means of stress test to identify exertion induced ischemia or exertion induced left ventricular diastolic dysfunction [2, 3]. Furthermore, HG has been used in combination with dobutamine stress echocardiography to identify patients with MR who would probably benefit from percutaneous mitral valve repair [4]. However, there is no existing data describing the effect of isolated HG on the severity of MR.

MR is the most common valvular heart disease and represents the second most common indication for valvular heart surgery [5, 6]. In patients with heart failure, MR is associated with an adverse prognosis [7]. Therapeutic options are increasing due to evolving of transcatheter techniques for MR treatment [8, 8]. Therefore, a precise evaluation of the impact of MR on clinical symptoms is essential. Exercise echocardiography has been described to improve assessment of MR severity [9, 10]. It may also be used to predict outcome in patients with heart failure and mitral regurgitation, as a large exercise-induced increase in MR is associated with higher morbidity and mortality [11]. However, performing an echocardiographic study during dynamic exercise such as running on a treadmill or cycling on ergometer may not always be feasible [12, 13]. The effect of HG exercise as an easy to perform method has not yet been systematically evaluated in patients with MR.

In this single-center study, we examined the effect of HG on MR in a large cohort of patients with different pathologies and severities of the disease. We aimed to investigate a possible role of HG in the echocardiographic evaluation of MR.

## Methods

### Study population

During the recruiting period, we screened all patients who received transthoracic echocardiography (TTE) in our laboratory. Every patient who was found to have MR, aside of its pathology or severity, and was sufficiently able to perform dynamic HG exercise was included. After completing the echocardiographic study at rest, the patient was requested to perform dynamic HG exercise. During the exercise, images of mitral regurgitation were acquired and saved for later evaluation. Due to the difficulty in applying the proximal isovelocity surface area (PISA) method for MR quantification in patients with a previous intervention on the mitral valve (percutaneous or surgical), those patients were excluded from the final analysis. Furthermore, patients in whom the use of PISA method for MR quantification was not suitable (e.g. very eccentric jet or multiple jets) were also excluded. Finally, we included 722 patients with different severities of MR.

### Transthoracic echocardiography

Echocardiographic studies were performed with patients lying in left decubitus position. Examinations were performed using commercially available echocardiographic system (Vivid E9. General Electric Vingmed. Horten. Norway) and 2D transthoracic probe (M5S. General Electric Vingmed. Horten. Norway). Echocardiographic examinations were performed according to recommended standards of the European association of echocardiography [14]. In patients with atrial fibrillation, all echocardiographic measurements were done over at least three cycles and the average value of these measurements was determined to minimize variability. Apical 4-chamber view was used for calculation of effective regurgitant orifice area (EROA) and the regurgitant volume (RVOL) using the PISA method. Maximal instantaneous regurgitant flow was calculated as 2*π*r^2^* v _aliasing_, where r was the maximal distance to the contour of aliasing velocity v_aliasing_ with a hemispheric contour assumed. EROA was obtained by dividing maximal instantaneous regurgitant flow by peak regurgitant velocity obtained by continuous-wave Doppler [15]. We calculated EROA and RVOL of MR at rest and during HG. Increase of MR was defined as any increase in EROA or RVOL during dynamic HG exercise.

Other echocardiographic parameters such as left ventricular ejection fraction (LV-EF), left atrial area (LA), systolic pulmonary artery pressure (sPAP) and tricuspid annular plane systolic excursion (TAPSE) were calculated. The association between these parameters and the effect of HG on MR was assessed.

### Handgrip technique

After completing the echocardiographic examination at rest, patients were given a small, elastic, hand-sized training ball in each hand and they were instructed to perform dynamic HG exercise while still lying in left decubitus position. Patients were advised to contract and open their hands in a continuous manner to squeeze and release the ball repeatedly. The exercise was regularly continued for 3 min.

### Additional information

Clinical characteristics of the patients including coronary artery disease, New York Heart Association (NYHA) dyspnea classification, the presence of current or any history of atrial fibrillation were obtained from the previous medical documents of the patients. Unless clinically indicated, coronary artery disease was not newly investigated in the context of this study. These characteristics were assessed to evaluate a possible association between these factors and the effect of dynamic HG on MR. NT-pro brain natriuretic peptide (NT-proBNP) level was also evaluated to identify a possible association between heart failure and the increase of MR during dynamic HG [16, 17].

### Data analysis

Clinical and echocardiographic data of the patients were retrospectively analyzed. We classified patients according to the etiology of disease into primary and secondary MR [18]. Analysis was carried out in all patients and in each of the two groups separately. Finally, to investigate if HG exercise would lead to an upgrade in classification of regurgitation severity from non-severe MR at rest to severe MR during HG, we performed an additional analysis. For this analysis, patients who already had severe MR at rest (patients with EROA ≥ 40 mm^2^, RVOL ≥ 60 ml in primary MR and EROA ≥ 20 mm^2^, RVOL ≥ 30 ml in secondary MR) were excluded [15, 19].

### Statistical analysis

All statistical analysis was performed with SPSS software (version 25, IBM Corp., Armonk, NY, USA) and MedCalc software (version 19.1; Mariakerke, Belgium).

Categorical variables were summarized as count (percentage) and continuous variables as mean ± standard deviation. Distributions of continuous variables were compared with t-test, association of categorical variables was assessed by Pearson´s chi-square test. To find variables for prediction of the increase in MR under HG univariate logistic regression analysis was performed. p values < 0.05 were considered significant.

## Results

Clinical characteristics and echocardiographic findings of the patients are summarized in Table 1. Among these patients, 132 were classified as primary MR (18%) and 590 as secondary MR (82%).

**Table 1:** Clinical characteristics and echocardiographic parameters.

Variable	All patients, n = 722	Primary MR, n = 132	Secondary MR, n = 590	p-value
**Clinical characteristics**				
Age, years	72 ± 12	68 ± 16	73 ± 10	0.001
Men, n	380 (52%)	44 (34%)	335 (57%)	< 0.001
CAD, n	490 (68%)	24 (18%)	466 (79%)	< 0.001
Atrial fibrillation, n	412 (57%)	63 (48%)	348 (59%)	0.051
NT-proBNP, pg/ml	3347 ± 3696	1275 ± 1310	3660 ± 3837	< 0.001
Hemoglobin, mg/dl	12.3 ± 1.8	13.4 ± 1.7	12 ± 1.8	0.025
Creatinine, mg/dl	1.3 ± 0.8	0.95 ± 0.2	1.5 ± 0.9	< 0.001
NYHA class				< 0.001
I	188 (26%)	59 (45%)	129 (22%)	
II	223 (31%)	36 (27%)	188 (32%)	
III	271 (38%)	32 (24%)	241 (41%)	
IV	37 (5%)	6 (4%)	32 (5%)	
Mean NYHA class	2.2	1.8	2.3	0.01
Beta-blocker, n	613 (85%)	91 (70%)	521 (88%)	0.01
Presence of HR increase during HG, n	527 (73%)	103 (78%)	432 (72%)	0.225
Mean increase of HR during HG, beat/min	6 ± 8	7 ± 8	6 ± 8	0.118
**Echocardiographic parameters**				
LV-EF, %	44 ± 13	58 ± 6	41 ± 12	< 0.001
LA area, cm^2^	26 ± 7	23 ± 7	26 ± 8	< 0.001
sPAP, mmHG	35 ± 12	32 ± 12	36 ± 11	0.005
TAPSE, mm	19 ± 5	21 ± 4	19 ± 5	< 0.001
EROA at rest, mm^2^	18 ± 9	18 ± 12	17 ± 8	0.324
EROA during HG, mm^2^	22 ± 11	22 ± 14	22 ± 10	0.870
ΔEROA, mm^2^	4 ± 6	3.5 ± 5	4 ± 6	0.261
RVOL at rest, ml	30 ± 9	31 ± 15	28 ± 12	0.260
RVOL during HG, ml	37 ± 17	38 ± 19	37 ± 16	0.676
ΔRVOL, ml	7 ± 9	6.5 ± 9	7 ± 9	0.426
LVEDd, mm	53 ± 9	48 ± 6	54 ± 10	< 0.001
LVEDs, mm	41 ± 12	33 ± 7	43 ± 12	< 0.001

*CAD* coronary artery disease

*EROA* effective regurgitant orifice area

*HG* handgrip

*HR* heart rate

*LA area* left atrial area

*LVEDd* left ventricular end-diastolic diameter

*LVEDs* left ventricular end-systolic diameter

*LV-EF* left ventricular ejection fraction

*NYHA* New York Heart Association

*RVOL* regurgitant volume

*sPAP* systolic pulmonary artery pressure

*TAPSE* tricuspid annular plane systolic excursion

### Effect of handgrip on heart rate

During dynamic HG exercise, mean heart rate (HR) increased from 72 ± 12/min to 78 ± 13/min. HR increased during HG in 527 patients (73%) while in 195 patients (27%) no increase of HR was observed. In patients with primary MR, HR increased from 69 ± 10/min to 76 ± 13/min (∆HR = 10.1%). In patients with secondary MR, HR increased from 72 ± 12/min to 78 ± 14/min (∆HR = 8.3%) (Table 1).

### Effect of handgrip on the severity of MR

**Fig. 1:**
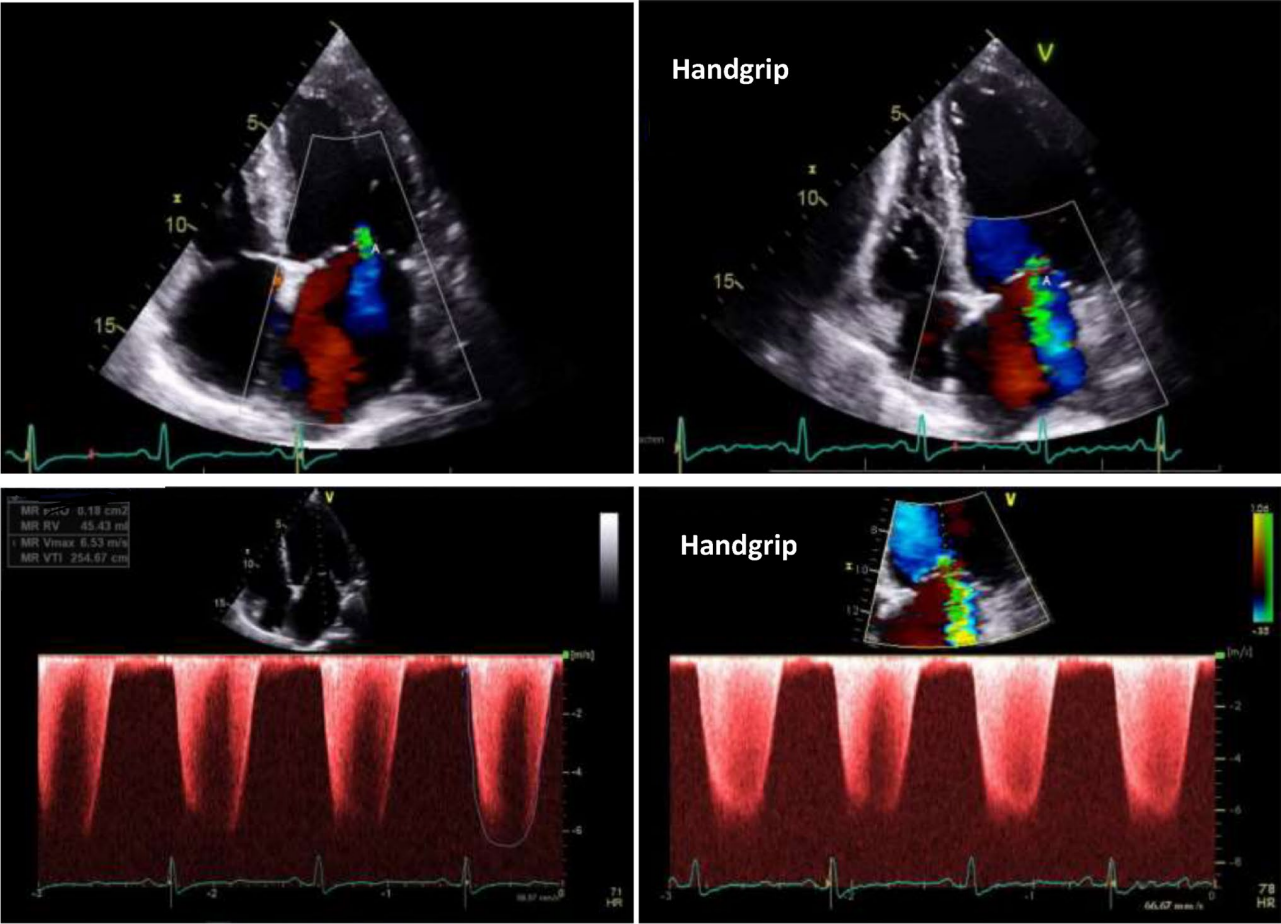
Baseline echocardiographic apical 4-chamber view with color Doppler (upper left) with continuous-wave Doppler assessment (lower left) in a patient with secondary mitral regurgitation (MR). There was an increase of MR (upper right) with denser continuous-wave Doppler signal during handgrip exercise (lower right)

An increase in MR during HG exercise, defined as any increase in EROA or RVOL, occurred in 390 patients (54%) (Fig. 1). In these patients, EROA increased by 4 ± 6 mm^2^ (25% of the baseline value) and RVOL by 7 ± 9 ml (27% of the baseline value). This increase was significantly associated with a larger baseline EROA at rest (OR 1.027, 95%-CI 1.008–1.045, p = 0.004) and with a greater baseline RVOL at rest (OR 1.015, 95%-CI 1.003–1.027, p = 0.012). It was also significantly associated with LA area (OR 1.025, 95%-CI 1.005–1.045, p = 0.014). In contrast, there was no association between the increase of MR during HG and any other echocardiographic parameters including LV dimensions or LV-EF. There was no association between this increase and clinical parameters including NYHA dyspnea class, NT-proBNP or atrial fibrillation (Table 2).

**Table 2:** Association between clinical variables and echocardiographic parameters and the increase of MR during HG in the study population

Variable	OR, 95%-CI	p-value
**Clinical variables**		
Age	1.008 (0.966–1.020)	0.211
Gender	0.920 (0.686–1.233)	0.576
Atrial fibrillation	0.988 (0.735–1.327)	0.934
NYHA functional classification (per class)	1.104 (0.936–1.302)	0.239
NT-proBNP	1.000 (1.000–1.000)	0.442
HR at rest	1.000 (0.989–1.012)	0.949
HR during HG	1.002 (0.991–1.013)	0.710
Increase of HR during HG	1.005 (0.298–4.358)	0.849
Beta-blocker	1.774 (1.170–2.688)	0.218
**Echocardiographic parameters**		
EROA at rest	1.027 (1.008–1.045)	0.004
RVOL at rest	1.015 (1.003–1.027)	0.012
LV-EF	0.995 (0.985–1.006)	0.411
LA area	1.025 (1.005–1.945)	0.014
sPAP	1.002 (0.989–1.015)	0.801
TAPSE	0.996 (0.968–1.024)	0.760
LVEDd	1.001 (0.995–1.027)	0.176
LVEDs	1.006 (0.991–1.023)	0.330

*EROA* effective regurgitant orifice area

*LA area* left atrial area

*LVEDd* left ventricular end-diastolic diameter

*LVEDs* left ventricular end-systolic diameter

*LV-EF* left ventricular ejection fraction

*NYHA* New York Heart Association

*RVOL* regurgitant volume

*sPAP* systolic pulmonary artery pressure

*TAPSE* tricuspid annular plane systolic excursion

### Differentiation between primary and secondary MR

**Fig. 2:**
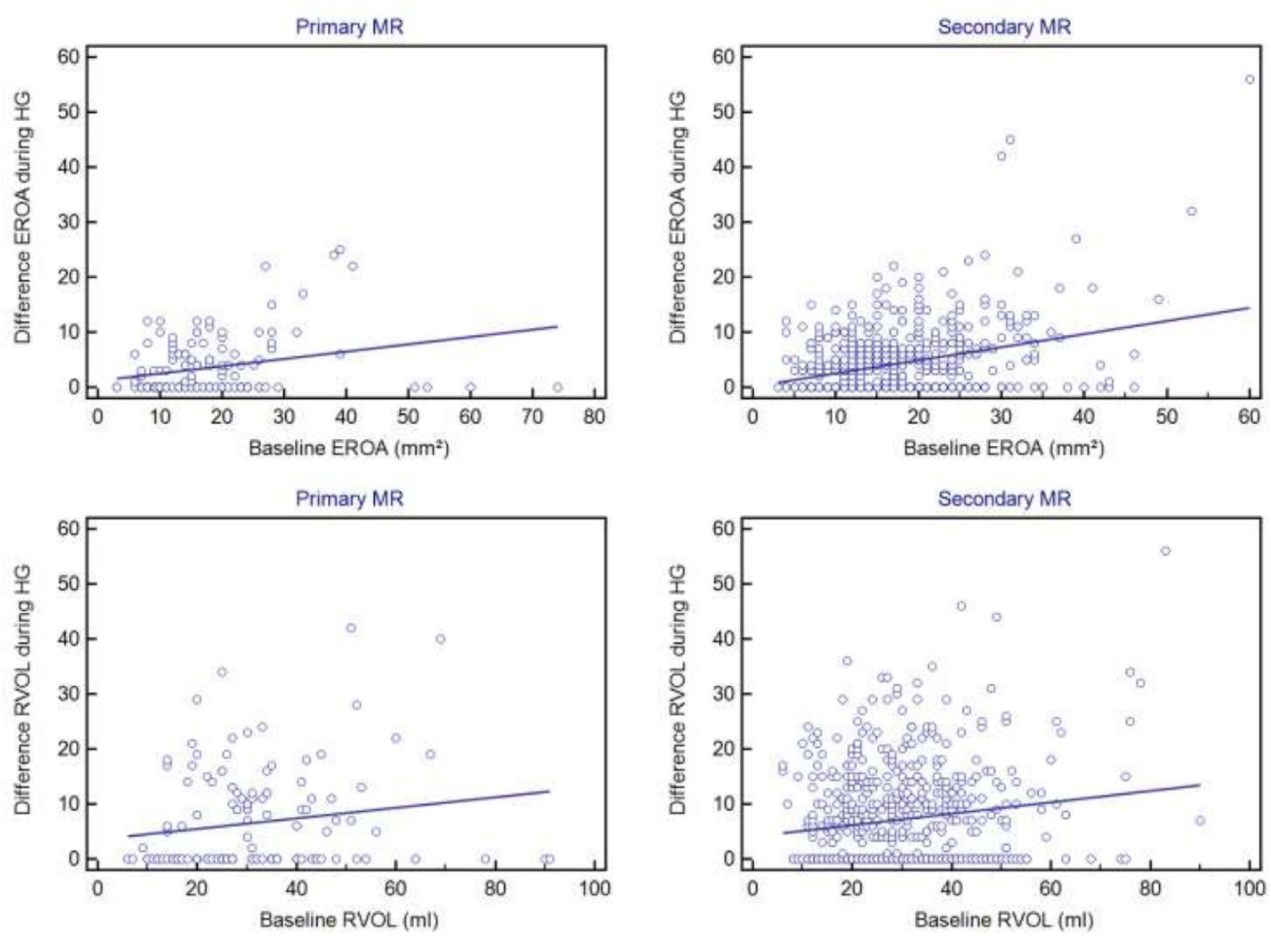
Baseline effective regurgitant orifice area (EROA) and its increase during handgrip (HG) exercise in patients with primary (upper left) and secondary mitral regurgitation (MR) (upper right) and corresponding baseline regurgitant volume (RVOL) and its increase during HG exercise in patients with primary (lower left) and secondary MR (lower right)

We classified the patients in two groups based on the etiology of MR. HG exercise increased regurgitation severity in 50% of patients with primary MR, and in 55% with secondary MR. The extent of this increase was not significantly different between patients with secondary MR (∆EROA = 4 ± 6 mm^2^, 26% of baseline value; ∆RVOL = 7 ± 9 ml, 28% of baseline value) and those with primary MR (∆EROA = 3.5 ± 5 mm^2^, 20% of baseline value; ∆RVOL = 6.5 ± 9 ml, 22% of baseline value) (p = 0.261 for ∆EROA and p = 0.426 for ∆RVOL; respectively). There was an association between this increase and LA size in patients with primary MR (OR 1.025, 95%-CI 1.005–1.945, p = 0.040) but not in those with secondary MR (OR 1.018, 95%-CI 0.997–1.040, p = 0.093). There was a significant association between the increase of MR during HG and baseline EROA and baseline RVOL at rest in patients with secondary (OR 1.038, 95%-CI 1.015–1.061, p = 0.001 and OR 1.015 95%-CI 1.001–1.028, p = 0.032; respectively) but not in those with primary MR (OR 1.008, 95%-CI 0.980–1.036, p = 0.593 and OR 1.018, 95%-CI 0.994–1.042, p = 0137; respectively). However, there was no association between this increase of MR during HG and any other echocardiographic parameters including LV dimensions or LV-EF. Furthermore, there was no association between this increase and clinical parameters including NYHA dyspnea class, NT-proBNP or atrial fibrillation in any of the two groups (Fig. 2, Table 3).

**Table 3:** Association between clinical variables and echocardiographic parameters and the increase in MR during HG in patients with primary and secondary MR

Variable	Primary MR		Secondary MR	
	OR, 95%-CI	p-value	OR, 95%-CI	p-value
Clinical variables				
Age (per year)	1.008 (0.987–1.029)	0.460	1.006 (0.991–1.021)	0.417
Gender (men)	1.157 (0.979–1.335)	0.950	0.933 (0.673–1.295)	0.680
Atrial fibrillation	1.095 (0.552–2.173)	0.796	0.953 (0.685–1.326)	0.775
NYHA functional Classification (per class)	0.923 (0.632–1.349)	0.680	1.128 (0.936–1.361)	0.266
NT-proBNP	1.000 (1.000–1.000)	0.872	1.000 (1.000–1.000)	0.450
Increase of heart rate during HG	0.495 (0.045–5.432)	0.565	1.946 (0.354–10.710)	0.444
Beta-blocker	2.033 (0.949–4.354)	0.066	1.610 (0.965–2.686)	0.068
Echocardiographic parameters				
EROA at rest (per mm^2^)	1.008 (0.980–1.036)	0.593	1.038 (1.015–1.061)	0.001
RVOL at rest (per ml)	1.018 (0.994–1.042)	0.137	1.015 (1.001–1.028)	0.032
LV-EF	1.084 (0.992–1.183)	0.073	0.995 (0.983–1.008)	0.450
LA area (per cm^2^)	1.056 (1.003–1.113)	0.040	1.018 (0.997–1.040)	0.093
sPAP (per mmHg)	1.010 (0.980–1.040)	0.526	0.999 (0.985–1.014)	0.933
TAPSE (per mm)	0.975 (0.906–1.048)	0.493	1.003 (0.972–1.035)	0.842
LVEDd (per mm)	1.057 (0.998–1.120)	0.557	1.005 (0.988–1.022)	0.582
LVEDs (per mm)	1.045 (0.990–1.103)	0.114	1.001 (0.988–1.015)	0.852

*EROA* effective regurgitant orifice area

*LA area* left atrial area

*LVEDd* left ventricular end-diastolic diameter

*LVEDs* left ventricular end-systolic diameter

*LV-EF* left ventricular ejection fraction

*NYHA* New York Heart Association

*RVOL* regurgitant volume

*sPAP* systolic pulmonary artery pressure

*TAPSE* tricuspid annular plane systolic excursion

### Effect of HG on the classification of MR severity

**Fig. 3:**
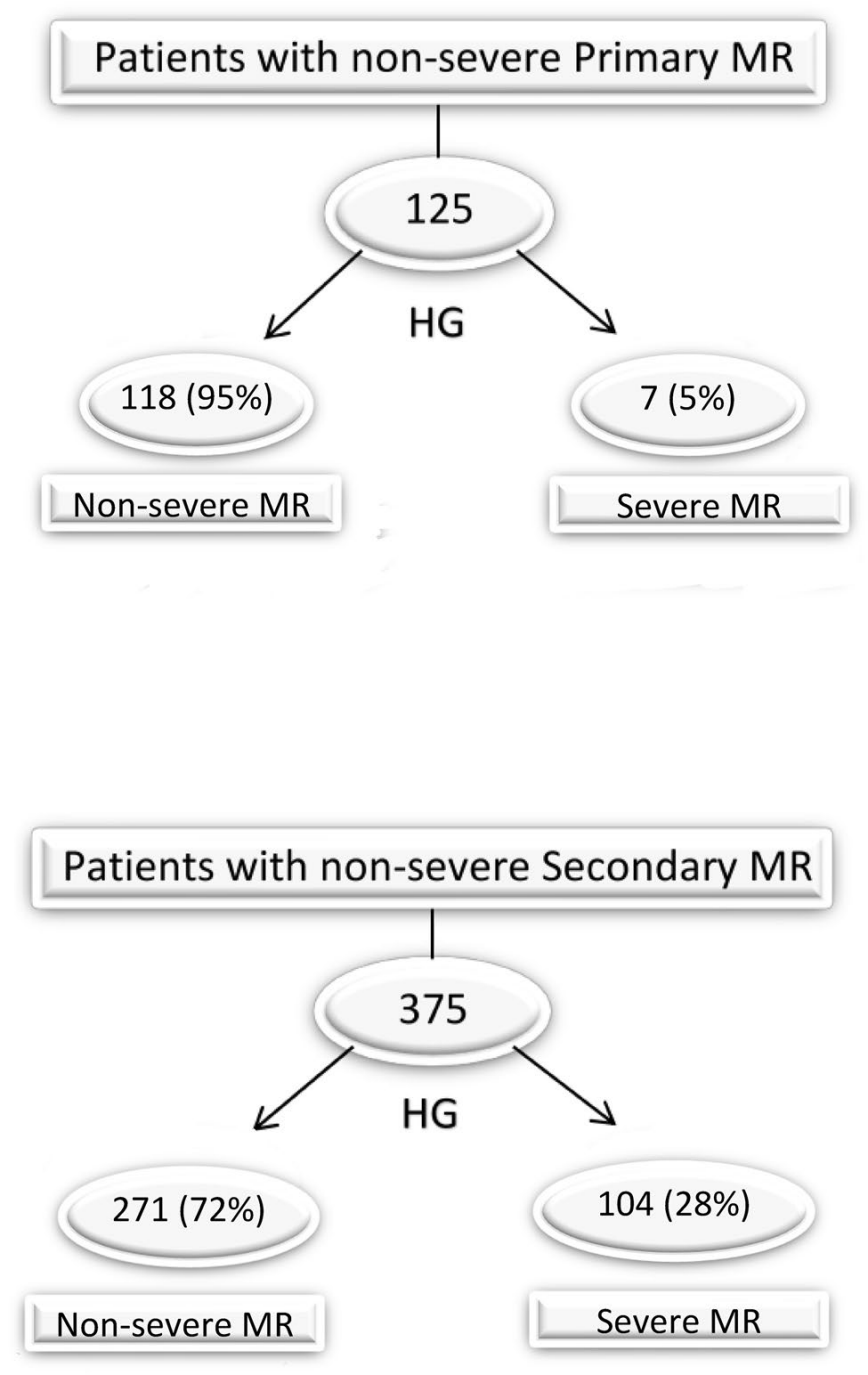
Effect of handgrip (HG) exercise on the classification of regurgitation severity in primary and secondary mitral regurgitation (MR)

In order to evaluate if HG exercise may lead to a reclassification of MR severity from non-severe MR at rest to severe MR during HG, we excluded patients who already had severe MR at rest for this additional analysis. In patients with primary disease, MR was classified as non-severe (EROA < 40 mm^2^, RVOL < 60 ml) in 125 patients at rest. In 7 of these patients (5%) there was an increase from non-severe to severe MR (EROA ≥ 40 mm^2^, RVOL ≥ 60 ml) during dynamic HG (Fig. 3). There was a significant association between baseline EROA and RVOL and this upgrade in the classification of MR severity during HG. However, there was no association between this upgrade in the classification of MR severity and other clinical or echocardiographic parameters in primary disease (Tables 4, 5).

**Table 4:** Comparison of clinical characteristics and echocardiographic parameters between subgroups of patients with non-severe MR at rest and during HG and patients with non-severe MR at rest and severe MR during HG

Variable	Non-severe MR at rest and during HG	Non-severe MR at rest and severe MR during HG	p-value	Non-severe MR at rest and during HG	Non-severe MR at rest and severe MR during HG	p-value
Pathology	Primary	Primary		secondary	secondary	
Number of patients	118	7		271	104	
Clinical characteristics						
Age, years	68 ± 17	67 ± 24	0.934	73 ± 11	75 ± 10	0.272
Men, n	37 (32%)	4 (57%)	0.158	134 (49%)	51 (49%)	0.944
Atrial fibrillation, n	55(47%)	4 (57%)	0.588	146(54%)	64 (61%)	0.242
NYHA functional classification, n			0.256			0.020
I	54 (47%)	2 (28%)		81 (30%)	17 (16%)	
II	33 (28%)	1 (14%)		90 (33%)	33 (32%)	
III	26 (22%)	3 (44%)		88 (33%)	49 (47%)	
IV	4 (3%)	1 (14%)		11 (4%)	5 (5%)	
Beta-blocker, n	80 (68%)	5 (71%)	0.854	232 (86%)	98 (94%)	0.026
Presence of HR increase during HG, n	93 (79%)	5 (71%)	0.645	195 (72%)	75 (72%)	0.975
Mean increase of HR during HG, (beat/min)	7 ± 8	10 ± 13	0.357	6 ± 7	6 ± 8	0.902
Echocardiographic parameters						
LV-EF, %	58 ± 6	60 ± 2	0.362	43 ± 13	40 ± 12	0.059
LA area, cm^2^	23 ± 7	26 ± 7	0.303	25 ± 8	25 ± 7	0.882
sPAP, mmHg	32 ± 12	38 ± 18	0.433	35 ± 12	35 ± 11	0.799
TAPSE, mm	21 ± 5	20 ± 7	0.807	19 ± 6	19 ± 5	0.211
EROA at rest, mm^2^	15 ± 6	34 ± 5	< 0.001	12 ± 4	15 ± 2	< 0.001
EROA during HG, mm^2^	18 ± 7	51 ± 9	< 0.001	14 ± 4	23 ± 4	< 0.001
RVOL at rest, ml	28 ± 11	52 ± 10	< 0.001	22 ± 8	26 ± 6	< 0.001
RVOL during HG, ml	34 ± 14	74 ± 15	< 0.001	26 ± 8	39 ± 9	< 0.001
LVEDd, mm	48 ± 6	54 ± 7	0.231	54 ± 10	52 ± 11	0.850
LVEDd, mm	32 ± 7	37 ± 3	0.129	44 ± 12	43 ± 2	0.473

*EROA* effective regurgitant orifice area

*LA area* left atrial area

*LVEDd* left ventricular end-diastolic diameter

*LVEDs* left ventricular end-systolic diameter

*LV-EF* left ventricular ejection fraction

*NYHA* New York Heart Association,

*RVOL* regurgitant volume,

*sPAP* systolic pulmonary artery pressure

*TAPSE* tricuspid annular plane systolic excursion

**Table 5:** Association between clinical variables and echocardiographic parameters and the upgrade in MR severity calssification from non-severe MR at rest to severe MR during HG in patients with primary and secondary MR

Variable	Primary MR		Secondary MR	
	OR, 95%-CI	p-value	OR, 95%-CI	p-value
Clinical variables				
Age (per year)	0.998 (0.955–1.043)	0.934	1.012 (0.991–1.034)	0.272
Gender (Men)	0.343 (0.073–1.609)	0.175	1.034 (0.651–1.610)	0.918
Atrial fibrillation	1.527 (0.327–7.124)	0.590	1.316 (0.830–2.084)	0.243
NYHA functional Classification (per class)	1.947 (0.872–4.341)	0.104	1.486 (1.138–1.940)	0.004
NT-proBNP (per 100 pg/nl)	1.007 (0.821–1.236)	0.947	1.070 (0.992–1.153)	0.078
Increase of heart rate during HG	0.672 (0.123–3.672)	0.646	1.008 (0.609–1.668)	0.975
Beta-blocker	1.187 (0.220–6.401)	0.842	2.675 (1.096–6.533)	0.031
Echocardiographic parameters				
EROA at rest (per mm^2^)	2.485 (0.999–6.184)	0.050	1.308 (1.209–1.415)	< 0.001
RVOL at rest (per ml)	1.239 (1.090–1.409)	0.001	1.065 (1.032–1.100)	< 0.001
LV-EF	1.277 (0.790–2.066)	0.318	0.983 (0.966–1.001)	0.060
LA area (per cm^2^)	1.055 (0.952–1.170)	0.303	1.002 (0.973–1.032)	0.881
sPAP (per mmHg)	1.033 (0.979–1.090)	0.235	0.798 (0.983–1.023)	0.798
TAPSE (per mm)	0.980 (0.834–1.151)	0.805	0.970 (0.925–1.017)	0.210
LVEDd (per mm)	1.161 (1.015–1.328)	0.209	0.998 (0.976–1.020)	0.849
LVEDs (per mm)	1.098 (0.975–1.217)	0.131	9.996 (0.977–1.014)	0.635

*EROA* effective regurgitant orifice area

*LA area* left atrial area

*LVEDd* left ventricular end-diastolic diameter

*LVEDs* left ventricular end-systolic diameter

*LV-EF* left ventricular ejection fraction

*NYHA* New York Heart Association

*RVOL* regurgitant volume

*sPAP* systolic pulmonary artery pressure

*TAPSE* tricuspid annular plane systolic excursion

In patients with secondary disease, MR was classified as non-severe (EROA < 20 mm^2^, RVOL < 30 ml) in 375 patients at rest. In 104 of these patients (28%) there was an increase from non-severe to severe MR (EROA ≥ 20 cm^2^, RVOL ≥ 30 ml) during HG (Fig. 3). There was a significant association between baseline EROA and RVOL and this upgrade in the classification of MR severity during HG. Furthermore, there was a significant association between the severity of exertional dyspnea (NYHA class) and this upgrade in the classification of MR severity (OR 1.486, 95%-CI 1.138–1.940, p = 0.004). Moreover, intake of beta-blocker was more common in patients with upgrade in the classification of MR severity than in patients without this increase (94% vs 86%; p = 0.026). There was no association between this upgrade in the classification of MR severity and any other clinical or echocardiographic parameters in secondary disease (Tables 4, 5).

## Discussion

The major findings of this study are (1) dynamic HG exercise increases MR in about half of patients independent of the etiology, (2) the absolute increase of MR during HG is significantly associated with larger baseline EROA and RVOL in patients with secondary MR and with more dilated LA in those with primary MR, (3) dynamic HG exercise changes the classification of MR severity from non-severe MR at rest to severe MR during HG in about one third of patients with secondary MR with a significant association between this reclassification and the severity of exertional dyspnea in this subgroup.

General cardiovascular response to exercise has been described in detail [20]. However, this response may vary according to the type of exercise. Cardiovascular response to static exercise such as static handgrip or weightlifting is different from response to dynamic exercise such as swimming or running. Dynamic exercise causes a relevant increase in heart rate, cardiac output, and oxygen consumption with lesser effect on blood pressure. In contrast, isometric exercise causes mainly an increase in blood pressure with lesser effects on other parameters [21]. HG exercise is further divided into static, also known as sustained HG, where the hand is continuously and constantly contracted against a given resistance, and dynamic HG, also known as repeated hand-squeeze where the hand is contracted and opened continuously. Louhevaara et al. compared the cardiorespiratory response to each type of HG exercise and did not find any difference regarding blood pressure, heart rate or ventilatory gas exchange [23]. The extent of these hemodynamic effects of HG is determined by the intensity of the contraction and its duration [24]. In patients with primary MR, there is only limited data regarding the role of exercise echocardiography [25]. However, the effect of exercise on secondary MR is more investigated. Because the response to exercise in patients with cardiomyopathies is different from normal subjects, exercise leads to a volume and pressure overload on the left ventricle, causing its dilation and consequently an increase in secondary mitral regurgitation. This effect was observed in several studies [26]. Regarding static exercise, Keren and colleagues reported that static HG caused a significant increase in the severity of secondary MR in 17 patients with advanced heart failure [27]. However, this study was performed before the era of modern medical treatment or device-assisted heart failure therapy, which are known to improve MR in patients with heart failure [26]. The role of repeated hand-squeeze or dynamic handgrip exercise as a mix between static and dynamic exercise on MR is not known.

Our study showed that dynamic HG exercise increased mitral regurgitation in some patients. Nonetheless, this effect and its extent were extremely variable among the study population. Comparing these results to the study of Lancellotti et al., which examined the impact of dynamic exercise (bicycle exercise) on the severity of secondary MR, we saw a qualitatively similar, but quantitatively lesser effect of dynamic HG on MR compared to dynamic exercise [11]. In that study, a mean increase in EROA of 8 ± 10 mm^2^ during exercise was noticed, compared to a mean increase in EROA of 4 ± 6 mm^2^ during dynamic HG in our study. A variable effect of dynamic exercise on MR was also seen, as EROA decreased in some patients. Furthermore, our results showed that there was quantitatively more increase of regurgitation during HG in patients with more severe MR with larger baseline EROA and RVOL at rest, which was also comparable to the results from Lancellotti et al. [11], as EROA increased by ≥ 13 mm^2^ in 48 of 51 patients with severe secondary MR (EROA ≥ 20 mm^2^) at rest in their study. However, this comparison was only intended to give an idea about the similar effects of dynamic exercise and dynamic HG on MR and does not replace a head-to-head comparison of the two methods.

In clinical practice, the absolute increase of MR during HG may not play a major role but rather the exercise induced change in the classification of MR severity. According to the recent guidelines, severe MR at rest, in presence of other findings, is an indication for intervention [19]. But change in the classification of MR severity during dynamic HG may particularly be important in symptomatic patients with non-severe MR at rest. Our study showed that dynamic HG changed the classification of regurgitation severity from non-severe MR at rest to severe MR in about one third of patients with secondary MR. This effect was especially seen in symptomatic patients with exertional dyspnea and it was significantly associated with the severity of exertional dyspnea (NYHA class). This finding implies that dynamic HG exercise during echocardiography may identify patients with non-severe secondary MR at rest, which may turn to severe MR during exertion. This is an important result and it may be the cornerstone for prospective studies assessing the prognostic importance of this finding and its role in deciding further management. The association between HG induced reclassification of MR severity and baseline EROA and RVOL is reasonable, as only less increase of these values is needed to upgrade the classification of regurgitation severity in patients with borderline MR at rest. As mentioned before, the effect of exercise on primary MR is not well established. Our results showed that dynamic HG had a similar effect on regurgitation in both primary and secondary MR, as MR increased by 20–28% in about half of the patients in both groups. However, in patients with primary MR, dynamic HG leaded to an upgrade in the classification in MR severity to be severe during HG in only 5% of patients. Nonetheless, it should be mentioned that dynamic HG in patients with primary MR led to an increase in mean EROA from 18 ± 9 mm^2^ to 22 ± 11 mm^2^ (Table 1). This change led to a conversion of classification from mild to moderate MR in patients with primary disease. It should be emphasized, that there was small subgroup of patients with primary MR (5%), who developed severe MR during HG (Table 5). In this subgroup baseline EROA was 34 ± 5 mm^2^, which meant MR was already moderate at rest. These individuals were statistical outliers within the complete sample of patients with primary MR, who had a mean EROA of 18 ± 9 mm^2^ at rest. However, as mentioned before, the role of exercise test in the evaluation of primary MR is not well established and should be further investigated.

A major constraint on the implementation of HG as a stress test in clinical routine might be the inconsistent intensity of contractions applied by different patients. The intensity of HG is usually measured as a percentage of the maximal voluntary contraction, which is a very subjective factor and is variable among patients [26-29]. Therefore, this intensity is expected not to be the same in the heterogeneous group of patients with MR. This may explain the variable effect of HG on MR in our study population.

It is common to withdraw beta-blockers before an exercise stress test. In our study population, 85% of patients were taking beta-blockers due to different indications. Although an increase in MR during HG was not significantly associated with a parallel increase in heart rate, as mentioned above, it cannot be ruled out that beta-blockers may have reduced the hemodynamic responses to HG and consequently its effect on the severity of MR. The finding, that intake of beta-blocker was associated with an upgrade in the classification of MR severity during HG in patients with secondary MR must be interpreted with caution. First, there was no significant association between intake of beta-blocker and the increase in MR during HG as shown in Tables 2 and 3. Secondly, in patients with secondary MR, there was a large difference in sample size between those with (n = 521) and without beta-blocker (n = 69). A further study to evaluate the physiologic cardiovascular response to dynamic HG exercise in patients taking beta-blockers with and without withdrawing this medication may be of interest.

Conclusively, our study proves that dynamic HG, like dynamic exercise, increases MR to a variable extent in some patients and it therefore might be used in echocardiographic evaluation of MR, especially in symptomatic patients with moderate MR at rest.

### Limitations

Although this is the first study to describe the effect of dynamic HG as a simple, bedside exercise method on MR in a large cohort of patients, we acknowledge that it has some limitations. The major limitation of this study is the lack of prognostic outcome data in patients with HG-induced increase in MR as well as the lack of the data comparing the effect of HG against the effect of dynamic exercise on the severity of MR. However, we believe that the results of this study may represent the basis for further research in this field. Another limitation is that classification of MR severity was only based on measurements of EROA and RVOL. Although quantification regurgitation severity using the PISA method remains the most recommended way to evaluate MR, this method may not be feasible in all patients. Furthermore, changes in blood pressure during HG were not reported. Finally, except for beta-blocker, the medical therapy of the patients was not described.

## Conclusions

Dynamic HG exercise, as an easy to perform method during echocardiography, increased regurgitation severity in about half of patients with MR independent of the etiology. In patients with secondary MR, HG exercise led to a reclassification of regurgitation severity from non-severe MR at rest to severe MR during HG in about one third of the patients. This effect was more evident in patients with exertional dyspnea and corresponded significantly to the NYHA dyspnea class. Therefore, dynamic HG may be used to identify exercise induced severe MR in symptomatic patients with moderate MR at rest.

## Abbreviations

EROA: Effective regurgitant orifice area
HG: Handgrip
HR: Heart rate
LA: Left atrial
LV-EF: Left ventricular ejection fraction
LVEDd: Left ventricular end-diastolic diameter
LVEDs: Left ventricular end-systolic diameter
MR: Mitral regurgitation
NT-proBNP: NT-pro brain natriuretic peptide
NYHA: New York Heart Association
PISA: Proximal isovelocity surface area
RVOL: Regurgitant volume
sPAP: Systolic pulmonary artery pressure
TAPSE: Tricuspid annular plane systolic excursion
TTE: Transthoracic echocardiograp

## References

[1] McCraw DB, Siegel W, Stonecipher HK, Nutter DO, Schlant RC, Hurst JW (1972). Response of heart murmur intensity to isometric (handgrip) exercise. Br Heart J.

[2] Dattilo G, Patanè S, Zito C, Lamari A, Tulino D, Marte F, Carerj S (2010). Handgrip exercise associated with dobutamine stress echocardiography. Int J Cardiol.

[3] Jake Samuel T, Beaudry R, Haykowsky MJ, Sarma S, Park S, Dombrowsky T, Bhella PS, Nelson MD (2017). Isometric handgrip echocardiography: a noninvasive stress test to assess left ventricular diastolic function. Clin Cardiol.

[4] Velu JF, Baan J, de Bruin-Bon HACM (2019). Can stress echocardiography identify patients who will benefit from percutaneous mitral valve repair?. Int J Cardiovasc Imaging.

[5] Nkomo VT, Gardin JM, Skelton TN, Gottdiener JS, Scott CG, Enriquez-Sarano M (2006). Burden of valvular heart diseases: a population-based study. Lancet.

[6] Enriquez-Sarano M, Akins CW, Vahanian A (2009). Mitral regurgitation. Lancet.

[7] Rossi A, Dini FL, Faggiano P, Agricola E, Cicoira M, Frattini S, Simioniuc A, Gullace M, Ghio S, Enriquez-Sarano M, Temporelli PL (2011). Independent prognostic value of functional mitral regurgitation in patients with heart failure. A quantitative analysis of 1256 patients with ischaemic and non-ischaemic dilated cardiomyopathy. Heart.

[8] Boekstegers P, Hausleiter J, Baldus S, von Bardeleben RS, Beucher H, Butter C, Franzen O, Hoffmann R, Ince H, Kuck KH, Rudolph V, Schäfer U, Schillinger W, Wunderlich N (2014). Percutaneous interventional mitral regurgitation treatment using the Mitra-Clip system. Clin Res Cardiol.

[9] Ansari MT, Ahmadzai N, Coyle K, Coyle D, Moher D (2015). Mitral valve clip for treatment of mitral regurgitation: an evidence-based analysis. Ont Health Technol Assess Ser.

[10] Lancellotti P, Garbi M (2018). Exercise stress echocardiography in degenerative mitral regurgitation. Circ Cardiovasc Imaging.

[11] Dulgheru R, Marchetta S, Sugimoto T, Go YY, Girbea A, Oury C, Lancelotti P (2017). Exercise testing in mitral regurgitation. Prog Cardiovasc Dis.

[12] Lancellotti P, Gérard PL, Piérard LA (2005). Long-term outcome of patients with heart failure and dynamic functional mitral regurgitation. Eur Heart J.

[13] Salden OAE, van Everdingen WM, Spee R, Doevendans PA, Cramer MJ (2018). How I do it: feasibility of a new ultrasound probe fixator to facilitate high quality stress echocardiography. Cardiovasc Ultrasound.

[14] Peteiro J, Bouzas-Mosquera A, Estevez R, Pazos P, Piñeiro M, Castro-Beiras A (2012). Head-to-head comparison of peak supine bicycle exercise echocardiography and treadmill exercise echocardiography at peak and at post-exercise for the detection of coronary artery disease. J Am Soc Echocardiogr.

[15] Evangelista A, Flachskampf F, Lancellotti P, Badano L, Aguilar R, Monaghan M (2008). European Association of Echocardiography recommendations for standardization of performance, digital storage and reporting of echocardiographic studies. Eur J Echocardiogr.

[16] Lancellotti P, Tribouilloy C, Hagendorff A, Popescu BA, Edvardsen T, Pierard LA, Zamoramo JL (2013). Recommendations for the echocardiographic assessment of native valvular regurgitation: an executive summary from the European Association of Cardiovascular Imaging. Eur Heart J Cardiovasc Imaging.

[17] Hill SA, Booth RA, Santaguida PL, Don-Wauchope A, Brown JA, Oremus M, Ali U, Sohel N, McKelvie R, Balion C, Raina P (2014). Use of BNP and NT-proBNP for the diagnosis of heart failure in the emergency department: a systematic review of the evidence. Heart Fail Rev.

[18] Maries L, Manitiu I (2013). Diagnostic and prognostic values of B-type natriuretic peptides (BNP) and N-terminal fragment brain natriuretic peptides (NT-pro-BNP). Cardiovasc J Afr.

[19] Dal-Bianco JP, Beaudoin J, Handschumacher MD, Levine RA (2014). Basic mechanisms of mitral regurgitation. Can J Cardiol.

[20] Baumgartner H, Falk V, Bax JJ, De Bonis M, Hamm C, Holm PJ, Lung B, Lancelotti P, Lansac E, Mu~noz DR, Rosenhek R, Sjögren J, Tornos Mas P, Vahanian A, Walther T, Wendler O, Windecker S, Luis Zamorano J,  (2017). 2017 ESC/EACTS Guidelines for the management of valvular heart disease. Eur Heart J.

[21] Laughlin MH (1999). Cardiovascular response to exercise. Am J Physiol.

[22] Lind AR, McNicol GW (1967). Muscular factors which determine the cardiovascular responses to sustained and rhythmic exercise. Can Med Assoc J.

[23] Motoi Y, Forton K, Pezzuto B, Fauro V, Naeiji R (2017). Resistive or dynamic exercise stress testing of the pulmonary circulation and the right heart. Eur Respir J.

[24] Louhevaara V, Smolander J, Aminoff T (2000). Cardiorespiratory responses to fatiguing dynamic and isometric hand-grip exercise. Eur J Appl.

[25] Lind AR, McNicol GW (1967). Circulatory responses to sustained hand-grip contractions performed during other exercise, both rhythmic and static. J Physiol.

[26] Picano E, Pibarot P, Lancellotti P, Monin JL, Bonow RO (2009). The emerging role of exercise testing and stress echocardiography in valvular heart disease. J Am Coll Cardiol.

[27] Bertrand PB, Schwammenthal E, Levine RA, Vandervoort PM (2017). Exercise dynamics in secondary mitral regurgitation: pathophysiology and therapeutic implications. Circulation.

[28] Keren G, Katz S, Gage J, Strom J, Sonnenblick EH, LeJemtel TH (1989). Effect of isometric exercise on cardiac performance and mitral regurgitation in patients with severe congestive heart failure. Am Heart J.

[29] Beyer SE, Sanghvi MM, Aung N, Hosking A, Cooper JA, Paiva JM, LEE AK., Fung K, Lujaschuk E, Carapella V, Mittelman MA., Brage S, Piechnik SK., Neubauer S, Petersen SE,  (2018). Prospective association between handgrip strength and cardiac structure and function in UK adults. PLoS ONE.

[30] Hartog R, Bolignano D, Sijbrands E, Pucci G, Mattace-Raso F (2018). Short-term vascular hemodynamic responses to isometric exercise in young adults and in the elderly. Clin Interv Aging.

[31] Lalande S, Sawicki CP, Baker JR, Shoemaker JK (2014). Effect of age on the hemodynamic and sympathetic responses at the onset of isometric handgrip exercise. J Appl Physiol (1985).

[32] Grucza R, Smorawiński J, Cybulski G, Niewiadomski W, Kahn JF, Kapitaniak BMonod H.  (1991). Cardiovascular response to static handgrip in trained and untrained men. Eur J Appl Physiol Occup Physiol.

